# Supplementary material for: Effect of Ambient Plasma Treatments on Thermal Conductivity and Fracture Toughness of Boron Nitride Nanosheets/Epoxy Nanocomposites
Source: Nanomaterials (Basel). 2022 Dec 27;13(1):138. doi: 10.3390/nano13010138 (PMC9823992; doi:10.3390/nano13010138)
Supplement: Supplementary file 1 [file nanomaterials-13-00138-s001.zip › nanomaterials-2099828-supplementary.pdf]

nanomaterials

Supplementary Materials

Effect of Ambient Plasma Treatments on Thermal Conductivity and Fracture Toughness of Boron Nitride Nanosheets/Epoxy Nanocomposites

Won-Jong Choi, Seul-Yi Lee * and Soo-Jin Park *

Department of Chemistry, Inha University, 100 Inharo, Incheon 22212, Republic of Korea
* Correspondence: sjpark@inha.ac.kr (S.-J.P.); leesy1019@inha.ac.kr (S.-Y.L.); Tel.: +82-32-876-7234 (S.-J.P.)

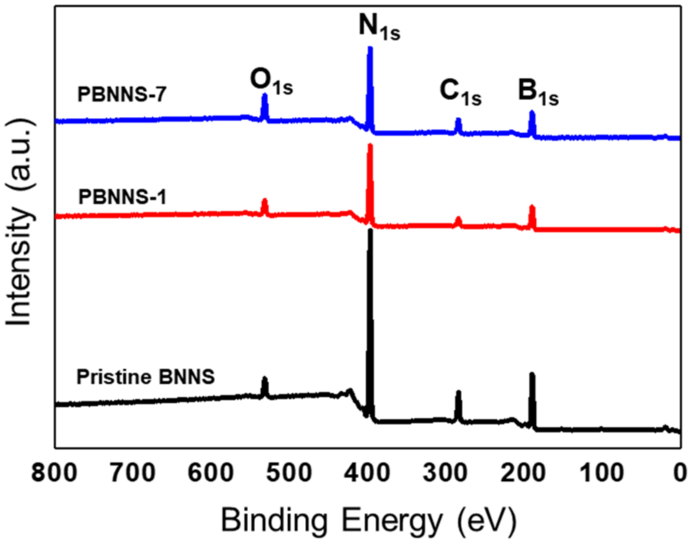

Figure S1. XPS spectra of pristine BNNS and PBNNS.

Table S1. XPS results of pristine BNNS and PBNNS.

Samples	Atomic (%)		
	B_{1s}	N_{1s}	O_{1s}
NEAT	54.09	42.25	3.62
P-BNNS 1	53.04	43.29	3.67
P-BNNS 3	53.68	42.11	4.21
P-BNNS 5	53.54	40.12	6.34
P-BNNS 7	53.43	38.05	8.52
P-BNNS 10	53.24	38.41	8.35

nanomaterials

MDPI

Table S2. Surface free energy, specific of the test wetting liquids used.

Wetting liquids	γ_L (mJ.m^{-2})	γ_L^L (mJ.m^{-2})	γ_L^{SP} (mJ.m^{-2})
Distilled water	72.80	21.80	0.38
Ethylene glycol	47.70	31.00	16.70
Diiodomethane	50.80	50.42	51.00

Table S3. The contact angles of PBE nanocomposites.

Specimens	Contact angle (θ)		
	Distilled water	Diiodomethane	Ethylene glycol
Neat	88.7 ±0.3	59.1 ±0.9	38.1 ±0.7
PBE-1	84.5 ±0.4	58.0 ±0.4	39.5 ±0.1
PBE-3	80.8 ±0.1	57.1 ±0.9	38.3 ±0.5
PBE-5	75.9 ±0.2	56.8 ±0.9	39.8 ±0.8
PBE-7	70.5 ±0.6	56.1 ±0.6	38.0 ±0.9
PBE-10	85.9 ±0.4	58.8 ±0.1	38.2 ±0.7

Table S4. Thermal conductivity parameter of PBE composites.

Specimens	Temperature °C	Diffusivity (mm^2.s^{-2})	C_p (J.gK^{-1})	Thermal conductivity (W.mK^{-1})
Neat	25	0.310	1.411	0.372
PBE-1	25	0.313	1.549	0.375
PBE-3	25	0.336	0.870	0.403
PBE-5	25	0.372	1.356	0.446
PBE-7	25	0.446	1.288	0.535
PBE-10	25	0.283	0.868	0.339
